# Gendered Perceptions of Sexual Behaviour in Rural South Africa

**DOI:** 10.1155/2011/973706

**Published:** 2011-07-03

**Authors:** C. Ndinda, U. O. Uzodike, C. Chimbwete, M. T. M. Mgeyane

**Affiliations:** ^1^Population Health, Health Systems and Innovation, Human Science Research Council, Pretoria 0001, South Africa; ^2^School or Politics, University of KwaZulu-Natal, Pietermartizburg 3209, South Africa; ^3^School of Public Health, University of Witwarsrand, Johannesburg 2000, South Africa; ^4^Independent Consultant, P.O. Box 50167, Umkhomazi 4170, South Africa

## Abstract

This paper discusses sexual behaviour findings collected through eleven homogenous focus group discussions conducted among women and men in a predominantly Zulu population in rural KwaZulu-Natal, South Africa. The objective of this paper is to shed light on sexual behaviour in a rural community. The findings suggest that sex is a taboo subject and the discussion around it is concealed in the use of polite language, euphemisms, and gestures. There are gender and generational dimensions to the discussion of sex. The contribution of this paper lies in the identification of what rural people discuss about sex and the influence of cultural practices and urban or global forces on sexual behaviour in rural areas. The paper adds to the growing body of literature on the use of focus groups in understanding sexual behaviour in rural contexts.

## 1. Introduction

This paper discusses findings on sexual behaviour collected through focus group discussions (FGDs). The objective is to report on views about sexual behaviour in a rural community in KwaZulu-Natal. The paper begins by providing the background literature dealing with previous research into sexual behaviour in using focus groups. We explain the context of the area where the study was conducted and proceed to report the findings on the community's discussion about sexual behaviour. The findings are followed by the discussion which pulls together key themes, and the conclusion recaps the key points arising from the paper.

## 2. Background

The existing literature on focus groups suggests that this technique has been used to collect data on a number of topics on sexual behaviour [1–3]. Yet, few studies have engaged critically with the suitability of focus groups in collecting sexual behaviour data [1]. 

In discussing premarital sexual behaviour among college students in Gujarat, Sujay [4] notes that the use of qualitative instruments such as focus groups, and in-depth interviews, revealed the levels of awareness with regard to sex education. Sujay [4] found that there was limited awareness on sexual and reproductive health matters thus highlighting the need for sex education to help youngsters adopt safe sex practices even at university level. The Gujarat study, using a combination of closed-ended questionnaires, focus groups, and in-depth interviews, noted that peer influences on sexual behaviour can be both positive and negative particularly for many youngsters whose source of information on sexual and reproductive health are their peers [4]. Peers also provide the social networks and supportive environment particularly when peer pressure results in romantic relationships. A key finding of the study was that indeed students in Gujarat are involved in romantic and sexual relationships [4]. However, findings from the qualitative techniques (focus groups and in-depth interviews) divulge the perceptions and attitudes towards the sexual relationships are gender biased. While most males accepted that premarital sex was acceptable if contraceptives were used, “Female students reflected more traditional attitudes towards premarital sex among both young men and women” [4]. Whereas it is generally tolerated that young men can be involved in premarital sex, young men do not accept to marry a girl who is a nonvirgin. Ironically young women do not use the same standards for young men and would generally accept a husband who has engaged in premarital sex.

A study on the factors that shape early sexual activity among adolescents in Jamaica noted that the motivating factors for early adolescent sexual activity were linked to poor economic conditions, peer pressure, of their gender identity, gendered inequalities, and power and the centrality of sexual behaviour to definitions of masculinity [5]. While the study confirmed that these factors often place adolescents in conflict with the dominant social ideals and values, it also noted that factors that shape adolescent sexual behaviour are also mediated by the adult family members, the church, school, and broader community. 

Buchanan-Aruwafu et al. [1] unravel how young people subvert language to discuss a taboo subject in a way that protects them from the harsh consequences of discussing a taboo subject—sex—openly. In exploring language and the mediation of desire in Malaita (Solomon Islands), Buchanan-Aruwafu et al. [1] allude to the strict code of conduct in Malaita to prevent people from discussing the sexual behaviours of others and the penalties imposed on those who make sexual propositions to members of the opposite sex. The code around sexual behaviour is so strict that public discussion of sex organs may still result in demands for compensation and violence from the male members of the female that is offended. A range of terminology is used to discuss sexual issues in a society where sex is a taboo subject. Polite terms are used to refer to female genitalia as an opening and to male genitalia as a long pointed stick [1]. The analyst notes that humour, metaphors, euphemisms, and slang are used by young people in public places to conceal their discussions on sexual matters. Thus, “Language is used both as an attempt to control sexual behaviours, to express desires for both married and unmarried youth, and to resist sexual prohibitions” [1]. The use of language with multiple meanings allows for the space to deny its meaning when confronted but also allows for shared meaning and understanding within the youth subculture. Given the scope of the paper, it explicates the language used to discuss sexual matters, but it does not delve in to sexual practices or what is done to enhance sexual pleasure. 

A study on young urban woman in Laos where focus groups were used to collect data on knowledge, attitudes, and practices related to sexual behaviour noted that most participants practiced vaginal sex which they referred to as “front door” sex. Vaginal sex was preferred because it indicates mutual respect and “it is “soft” and not violent” [6]. The study notes that young women are more likely to engage in longer sessions of sexual activity and “perform other sexual acts, including oral sex, anal sex (back door) and sex between the thighs” [6]. Pornography and peers were cited as sources of information about different styles of sexual activity. The study found that young women in Laos perform oral sex for their own pleasure and that of their partners as well as to avoid pregnancy. Furthermore, participants in the study reported that it was common for men to use their fingers to stiumlate the sexual organs of women [6]. 

The Laos study not only interviewed ordinary young women, but it also conducted focus groups with young commercial sex workers who noted that they often engaged in oral sex with older men to stimulate their erections before vaginal sex. Kissing and touching were also noted as techniques used to enhance sexual pleasure for the clients of the commercial sex workers [6]. The authors note that “nowadays many students have sex in exchange for money for their school fees, or materials without concern for what other people think” [6]. The notion of transactional sex emerges in this study where money features in steady relationships and where young girls prefer to have sex with older men that can buy them luxuries such as clothes, mobile phones, and motorcycles [6]. The study on young urban women in Laos sheds light on sexual practices, what is done to enhance sexual pleasure, attitudes of young women towards different sexual acts, and the transactional nature of sex. 

While studies on sexual behaviour among young people conducted in different contexts suggest young people are involved in premarital sex, there is consensus about the gendered expectations in terms of sexual behaviour and the double standards applied to men and women [4, 6]. Women are generally expected to remain chaste, but it is generally accepted that young men can have premarital sex. Writing with regard to Laos, Keobounphanh and Toole [6] notes that “A woman who retains her virginity until marriage is seen as a woman who is trusted and respected by men and admired by society and the family” [6]. In the same vein, Sujay [4] notes that although premarital sex is common in Gujarat, young men will always seek out virgins when they want to marry. The emphasis in the existing studies has been on understanding the sexual behaviour and reproductive health of young people with a few discussing sexual behaviour in the general population.

## 3. Background Context

The study described in this paper was conducted in KwaZulu-Natal province of South Africa within a rural African population typical of other areas in South Africa. Much of the area in this region is under the traditional authority of chiefs known as *Amakhosi* (singular term is *inkosi*), and while residents in each chiefdom owe allegiance to the local chief, they do not necessarily belong to the same clan. Most of the people in this region consider themselves Christians with the majority identifying with the African Independent Churches such as the Zionists and Shembe. The population of this area is about 90,000 people who live in 11,000 households [7] in the deep rural and periurban areas. The average household size in the area is 7 members which is much higher than the average in the province (4.2 persons) [7]. About 35.2% of the households are femaleheaded [7]. Although the area is rural, households are not clustered in villages as in rural areas in sub-Saharan Africa. Within the rural area, less than 14% of the households have access to piped water, and about half have neither toilets nor pit latrine. However, the nearest town has all the trappings of modernity with piped water, sanitation, electricity, roads, and refuse removal [7]. The neglect of the rural tribal area may be explained by the apartheid Group Areas Act of 1950 that created White towns in rural areas that were fully serviced with basic infrastructural services and separated from tribal settlements by highways, cemeteries, or railways. 

The conjugal relationships in this area comprise of marriages or long-term cohabitation. Long-term cohabitation involves sexual partners who are members of the same household. Long-term conjugal relationships are common in this area like in the rest of the country and are recognised by the state. In this area, marriage is rare and less than 50% of women are married but cohabitation and single parent households are common [8]. The levels of unemployment in this area are high with only 26% of the economically active adults being employed, compared to 50% employment rate among those living in the urban area [7]. 

Focus group discussions (FGDs) were conducted among men and women aged between 15 and 49 years in the study area. Women were recruited from the immunization and family planning clinics. We sought and received permission to approach women at the clinic from the health authorities, and we invited women for FGDs whilst they were in the waiting room. Men were recruited using the snow ball sampling technique from the community; it was assumed that these represented the likely partners for the target population of women recruited for FGDs. The weakness in using this technique is that the sample might be biased as people tend to recruit their own friends or relatives. However, our fears of such biases were allayed when some of those invited did not show up; also, the findings show a diversity of views. In addition, we conducted at least two FGDs for each component of the study population. 

The FGDs were conducted in isiZulu by four Zulu-speaking research assistants (RAs), three of them are females, who had experience in demographic and ethnographic data collection. Each focus group was moderated by a facilitator and the note taker jotted notes and also ensured that the audiorecording was in progress. Taking notes was a back-up strategy to ensure that if the electronic recorders failed (and they did sometimes), the discussion would still be recorded. The RAs interchanged roles from one group to another, so that each had the opportunity to facilitate a discussion and also to take notes. We aimed to get between six to twelve participants, but in one group, members brought their friends along, and dismissing the extra members would have been considered impolite, so they were accommodated in the discussion. We ensured that the quality of data and information collected during the sessions was not compromised by enlightening the facilitators about the possible pitfalls if the group was large. 

The objective of the FGDs was to gather the views of the general community with regard to HIV awareness and sexual behaviour. Participants were asked whether people talk about sex with others, with whom they discuss, about sex, the specific issues that they discuss and whether there is anything that people do to enhance sexual pleasure. The discussions took between one hour and half and two hours. 

All interviews were recorded, transcribed, and translated, verbatim below each subsection of the Zulu transcript. To ensure the reliability and validity of the data, the transcribed Zulu texts were given to an independent researcher to translate in order to verify and confirm the data. Transcripts were coded by two of the authors independently, using the themes explored in the focus groups and our own theoretical interest as a guide. The coding and interpretation of the data was then compared for consistency. There was general consensus on the major themes. The authors discussed the coding further and reconciled any differences.

## 4. Findings

There were between seven to sixteen participants in each FGD with a total of 107 participants. The average age of the rural women was 32 while that of the periurban women was 25.4 years. For the rural men, the mean age was 26.8 years and that of the periurban men was 29 years. Employment among the women was 20% both among the rural and periurban women. However, among the urban men, employment was 52% compared to only 17% among the rural men. 

### 4.1. The Focus Group Process

During the discussion which was facilitated by a woman, the discussion progressed well until the questions on sexual behaviour were posed. Some of the men were heard mumbling, *Iengane iyasibelesela *(This babe is nagging). For the particular facilitator, it was not just enough for her to state her organization but rather the area where she came from and her clan. Surprisingly, they were not too concerned about her male counterpart or other female members of the team; it may be because the facilitator was moderating the discussion. As in many other focus groups, the participants reacted with shock and silence when the topic of sex was posed. In a men's group, some participants even commented, *Hawu*, *iyakhuluma lengane*! (This babe can talk!). Such comments were made because of the gender and age of the particular facilitator. The moderator noted, F4: *From the way they were talking they were surprised that a young woman could discuss such issues*. It is taboo for young people to discuss sex issues in public particularly with elderly people. Had it been a young man discussing, the level of surprise would not have been the same as it was with the young woman. 

The debriefing sessions also shed light on the group dynamics and the response of the participants to the questions. Of note is a men's group where the participants seemed very organised. After introducing the research team and the purpose of convening the discussion, the group began complaining about how research had been conducted in their area, and yet no cure for HIV/AIDS had been found. In two men's groups, the participants were angry that the research being conducted had not resulted in the provision of antiretroviral therapy. They said, “*We're tired of discussing now*”. In a women's group, the concern was that a lot of effort was being put in preventing transmission to children, and nothing was being done to care for their mothers. The explanations provided put the participants at ease, and the research team was able to proceed. However, this did not stop participants from making comments on things that they found shocking, for example, “*Ngeke ngitshwele umfazi*” (a woman cannot tell me) in response to whether women have the right to say “no” to sex. The comment needs to be understood in the context of this community where, culturally, a man proposes to a woman and if a woman tries to propose she is considered disgraceful and lacking in respect. In contrast to these patriarchal and conservative comments, a surprising finding is that some participants volunteered information about sexual behaviour without much prompting. The only exception was when the subject of anal sex was introduced.

### 4.2. Sexual Behaviour

#### 4.2.1. Taboo Subject

The discussion about sexual behaviour was conducted in a rural area amongst the Zulu, an ethnic group that is steeped in many traditional mores and values. The topic was approached cautiously as sex is a taboo subject and therefore not one of the things that people openly speak about. Matters around sex are discussed with the young when they reach puberty. For girls, elder women are assigned to teach the maidens about sexual matters in preparation for marriage. Older men too discuss matters of sex with young men when preparing them for their role in marriage. The changing contexts of these traditional arrangements were issues of deep reflection and concern amongst the FGD participants. A number of female respondents felt very disturbed by the changing cultural norms pertaining to sexual behaviour (p: participant):

P3: We are Zulus … [when] we grew up … you would *ukuqoma* (choose a lover), while a virgin … you were given an older female whose role is to give sex education (*iqhikiza*). Such women are part of the organizational structure of the Zulu society for preparing girls to be responsible and respectable women in terms of Zulu culture. We were informed … Not [what] is being done now … for the Zulu the “real the one” (sex) [is] for *crossing legs *(thigh sex). We grew up in those times … In our land we lived like that, not “this one” (sex) of today. It sickens me … If I explain all our ways you will say we had a good life,—(*she sounded disappointed with the sexual behaviour and the upbringing of the youth of today*) (Woman).

P6: Awu, to us the African nation freedom is still a big problem. We are shy to tell our children about sex, we see it as if we are teaching them promiscuity. It is difficult to tell your partner or to remind her that you want sex, or … to discuss your sex life with your partner (Man).

Parents do not discuss sex with their children which reinforces the notion of silence around the subject. It is such a taboo subject that even a discussion of one's sex life with an intimate partner is difficult.

#### 4.2.2. Conservative

Sex being a taboo subject is not referred to in plain terms. The sexual act is colloquially referred to as “hitting” or “beating” (*shaya*), a polite way of describing it both among men and women. Sex is not discussed in daily discourse and explains the resistance experienced by the FGD moderators from some groups. In one of the men's groups, participants were openly against discussing the subject in the presence of the female moderator even if a male counterpart accompanied her. The men said,

P3: If there could be a way to finish this, it should just be finished ….

Zulu men often exhibit significant levels of anxiety in discussing private sex matters in the presence of women. During the discussion, the men were heard to say, “*Umfazi, umfazi*” (a woman is a woman) and, by implication, should know her place in society where she is expected to be submissive to her husband. Some participants threatened to cut short the discussion and refused to say openly what they did to enhance sexual pleasure; instead preferring to say that “*the rest is for the bedroom*”. While such responses illustrate resistance to the subject, it needs to be noted that this was a unionized group that began the discussion and attempted to steer it towards issues that were of concern to them (wage increases, medical aid, and HIV treatment). The facilitator was therefore critical and effective in steering the group towards discussing sexual behaviour.

Unlike with the organized group, in other men's groups moderated by a female alongside her male counterpart, some men openly discussed what they did to enhance sexual pleasure without inhibitions, often referring to the facilitator as “sisi”, a *Zulunised* version of the English word “sister”. Participants noted that enhancing sexual pleasure not only involved things done in the short-term but also longterm actions such as paying bride wealth (*Lobolo*).

P11: I see that what can cause it to be nice is to have my person (partner) esengimlobolile (have paid lobolo [bride wealth paid by the man's family to that of the woman in proposition for marriage], marry her … (*P8 and P5 talking and laughing in the background*) … no one will say, “Ey you are staying [cohabiting], just staying with my child, you have not paid for her” To enhance it I marry her. The rest is for the bedroom (Man).

The payment of lobolo gives the man assurance that the woman would not decide one day to walk away without any consequences. The payment of *Lobolo *also gives the women assurance that the men in their lives are committed rather than casual (*makwapheni*). Casual partners are contrasted with regular partners (*umaqondana*) in which the relationship is long term and publicly acknowledged either through the payment of *lobolo* and bearing of children even if it does not result in marriage. These issues notwithstanding, and despite the open attempt by some men to discuss freely, it became clear that the subject of sex is a difficult one to discuss openly. This was despite participants being reminded that the discussions were about general sexual behaviour in the community.

#### 4.2.3. Acceptable Responses

When women spoke, it was with reference to what they do as a social category and the same happened with the men. That notwithstanding, the gender-based distinction was useful as it allowed the groups to speak more freely on culturally sensitive matters. For instance, women across the focus groups identified about eight items, which also doubled as virginal inserts, for use to enhance sexual pleasure. This was despite the fact that, at first, some participants insisted that nothing was used to enhance sexual pleasure. However, on further probing, the women explained:

P3: There are lots … perhaps because I am using the *Jehovah one *(Pure sex without use of any aids or additives). I am satisfied when my partner romances me. I hate when a person has a mark here (*pointing to the side of the neck—referring to the love bites*) [*they laughed loudly*] … I hate it. I like to romance as a human; not that I have to put things. If we have to talk about these things … there are *silver bullets*, and “*where will I sleep*” (these are pills). There is another one like a heap … they administer *enema *with and others induce vomit with it … P8: That one like meat … P4: Like a piece of fat meat … they say specific ocean plant (amahlanzolwandle). … P3: yes it is like meat (Man).

The extract provides an example of participants who initially gave what they considered “correct” answers (*the Jehovah one*) but who in the course of doing this also provided data regarding things done to enhance sexual pleasure. Traditionally among the Zulu, men use a concoction of herbs to administer enema, which is believed to detoxify the kidneys and clean up the rectal system, thus boosting their sexual performance and enhancing sexual pleasure for their women. The description of certain plants and how they are used to enhance sexual pleasure gave others a clue of what was being described (*amahlanzolwandle*). The mention of enema here and in men's discussions suggests that it is a common practice in this rural area. The response in this case and throughout the discussions remained gendered.

### 4.3. Gendered Responses

#### 4.3.1. Women's Views

In four out of the eleven discussion groups, snuff was cited as one of the things that women insert into the vagina to enhance their partner's sexual pleasure. Three of these groups comprised of women and one comprised of men. Snuff is believed to tighten the muscles of the vagina thereby making intercourse pleasurable for the male partner. While women believe tightening of the vagina enhances sexual pleasure for the man, the men's group was of the view that snuff is harmful.

P2: … there is someone I know (an aunt). She puts the Zulu Tobacco (snuff) … when she has (*lifting her hand as a sign of drinking*), she says she put it even here (pointing to the genital area). They mention this snuff … the sour stone (*i-alamu*) and dettol. They say when you have used it your partner cannot feel that you have … P2: That is why I have said that this issue is difficult to always open up on it; although there are some who are not shy to say what they use. F4: what do you mean when you say you have … [*bending the hand to the side under the armpit*] P2: if you have another partner (*casual*), your man (regular) cannot feel that you have … [*still not giving a straight answer*] (observation of the facilitator) … I meant that. Others openly say how they do it. F4: Do they say why they are doing this? P2: … to stay tasty (nice). Stay young and do not get old (in the genital area) (Woman).

P7: Others go to the *inyanga* (traditional healer) … there is some “*muthi*” that he will infiltrate into the cuts (*izingcabo*) … P3: Yes, they say he cuts you on the “*police*” (genital area) (Woman).

Participants used gestures, or symbols to pass across points that they could not verbalize, for example, bending the hand to the side under the armpit to denote a casual partner; and referring to the female genital as the “police”.

The conversation, accompanied by gestures and incomplete sentences and euphemisms, is highly coded and contains layers of meaning which are only unravelled through probing. Such usages of coded language, gestures, and sanitized terms are often so complex that they may conceal crucial aspects of what is being communicated, even to a Zulu speaker from outside this community.

A range of herbal concoctions commonly referred to as *imbiza *(concoction of traditional Zulu medicine used to treat different ailments), Zulu *Muthi *(general term for traditional medicine), are used to enhance sexual pleasure by women. These include certain *muthi* applied to incisions made on the skin of the vagina. “*Bhekaminangedwa*” (look at me only) is a concoction bought from the *Inyanga (*traditional healer). When covertly put in the food of the man, *bhekaminangedwa* is believed to cast a spell on him, thereby causing him to be sexually attracted only in the woman that uses *Bhekaminangedwa* on him. This is believed to prevent the man from having sexual relations with secret lovers (*omakwapheni *(plural for *umakwapheni* [casual partner]). *Makwapheni* loosely translated denotes “under the armpit”, and when something is put under the armpit it is generally hidden from public view. Secret lovers or casual partners are referred to as *omakwapheni*. Another herb used is *amahlazolwandle*, which is an amoeba-like plant collected from the ocean. The plant is boiled, filtered, and then taken like tea. It is believed to enhance sexual pleasure among women. Participants readily volunteered information on what was generally used in the community to enhance sexual pleasure but steered clear of admitting that they used these as individuals. 

In terms of genital hygiene, we sought to understand whether women in this community usually clean inside their vagina, when they do it, how they do it, why they do it, and what they use to clean the vagina. When posed to the men's groups, participants were categorical that such information would be best obtained from the women themselves.

P8: again it can be answered by them because there are things which they can never know which concerns us (Man).

Five out of the six women's groups provided information on vaginal inserts. The women suggested that the only thing used for cleaning the vagina was water and soap. Women argued it is important to use cold water as warm water damages the elasticity of the vagina. Only in one men's group did a few participants have an idea of the vaginal inserts used by women. In three out of six women's groups, Dettol was cited as being used for cleaning the vagina because it is believed to dry and tighten it thereby making sex pleasurable for the man.

In this area wetness is also believed to denote unfaithfulness by the woman. Not surprisingly, a woman may go to great lengths to ensure that she is dry. In two women's groups and one men's group, participants cited “*ialamu*”, a white stone with a sour taste that is bought in pharmacies, and normally used for treating mouth sores. However, participants noted that *ialamu* is used for enema or induced vomiting to clean up the system and make sex pleasurable among both women and men. *Blue Stone*, cited in three groups, two comprising of women and one of men, is crushed like sandstone and bought in shops. It comes in different colours and has some glitter and the smell of perfume. When applied in the vagina, Blue Stone is believed to tighten the vagina and makes sex pleasurable for the male partner. Vaginal inserts in this community have a dual role of genital hygiene as well as enhancing sexual pleasure. Examples are “*dubulageqe *(herb commonly used to detoxing among the Zulu)” which is used to detoxify the woman's entire system; it is said to enhance sexual pleasure for the woman as it removes obstacles to sexual pleasure such as bloatedness. The findings suggest that vaginal inserts have dual purpose for genital hygiene and also enhancing sexual pleasure and that knowledge on vaginal inserts and genital hygiene is gendered.

#### 4.3.2. Men's Views

Whereas only one women's group cited foreplay as one of the things done to enhance sexual pleasure, all of the five men's groups cited it as an important part of enhancing sexual pleasure. Among the women's group, foreplay included watching erotic movies to get them into the mood for intercourse. Like the women, the men noted that foreplay involved watching blue movies and reading erotic magazines. Unlike the women's groups, the men's groups cited different styles of sex as enhancing sexual pleasure. Those cited in three of the men's groups included oral sex and vaginal sex “dog style”.

P13: Love and to give a person her time; if you have placed me here and say I am yours and I say the same to you it is like there is no other person in this earth besides the two of us. Besides wanting many things (sex) then hold me nicely, touch me nicely and then you forget that you are a Shamase (surname). … You say I am yours and I also say you are mine after “*isithuthwane* (generally refers to fits or seizure but in this context the expression is used in a colloquial way to refer to orgasm)” (orgasm) has passed (Man).

P11**: **Oh! Maybe to … it depends on how strong you are or how weak you are … But what makes you to be nice is to be just strong. A person can say wake up and you say “hhie … hhie …” [*other participants laughed*] you cannot wake up you are dead asleep … If you have an enema you can go on without tiring. (Man).

A good amount of slang, euphemisms, and polite language were used in discussing sexual behaviour. Going to lengths to use sanitised language suggests that sex is a taboo subject, and when conducting research, it is important to use polite terms in order to understand sexual behaviour in rural KZN.

A distinction was made between things done culturally to enhance sexual pleasure and modern products bought in shops and pharmacies. The things done culturally to enhance sexual pleasure are described in the following extracts:

P11: Before there was “*izichonco*” (acupuncture) that functioned to make it nice if it was no longer nice. In our forefathers time there was *this thing *(traditional tablets) that they would put *there* (penis). Some other times they would drink it with milk or with soup … without telling you what it does but … it is like pills (term commonly used to refer to medicine). … (Man).

P8: the Zulu *muthi* is very strong in a woman … The woman, even if she has had sex before, if she has found the Zulu *muthi*, you would hear it being said that, “Ey, somebody is hot”. It is this Zulu *muthi*. In some other time it is smeared *here in front* by the men (on the penis), when I have come to you, you would forget even someone you have been with (Man).

Like the women, the men use traditional herbs generally known as* imbiza. *In urban centres,* Imbiza *is sold in the streets by *Muthi* traders who give guidelines on usage. These and other herbal concoctions can be purchased throughout South Africa at minibus terminals (taxi ranks), which are accessible and convenient as popular commuter points for the general public. Often purchased and used secretly, herbal concoctions are reputed to assist in sustaining erections. Beyond herbal medicines, men also resort to other traditional ways of enhancing sexual pleasure such as having enema, mentioned in four out of the eleven groups. Doing enema is believed to help men not only to stay strong during intercourse but also to sustain erection, thus enhancing pleasure for the woman.

Three out of the five men's groups noted that over-the-counter medication (*amaphilisi*—in Zulu—is a general term for tablets) can also help to enhance sexual pleasure among men. Those cited include *impotex *which is believed to help sustain an erection and prevent early ejaculation. In two men's and two women's groups, participants cited “silver bullets” used by both men and women to enhance sexual pleasure. Among the women, silver bullets are used to reduce water in the vagina thereby making sex more pleasurable for the men. “Dry” sex is considered pleasurable in this region, hence a range of vaginal insertions are used to ensure that the vagina remains dry.

P8: Among the pills there is *tiger*, *tingdon* is smeared here in *ushemula* (chimney) (penis) in the beginning. And when you *go there *(*sexual act*),… the person you love just feels something she has never felt before (#9 there is just a big heater) (Man).

P7: Awu my brother there is something else and pills to make the *stick *… because sometimes while you are doing sex … the *stick* just falls but the female has not *arrived there* [*reached orgasm*]. There are pills that make you strong, and your *stick* not to fall … yes there are … *bullet*, and *power up*, you see pills like those? … #6: *Power rise*! (Men).

Products bought in shops and pharmacies featured among the things that men used to enhance sexual pleasure. Two of the men's groups noted that silver bullets helped to sustain an erection much longer, thus making sex more pleasurable for their female partners. In one of the men's groups, participants also cited “*power rise*” as one of the tablets that they use to ensure sustained erection. In the same group, “*importex*” was cited as being useful in preventing early ejaculation, thus making sex pleasurable for the woman.

## 5. Discussion: Understanding Sexual Behaviour

Sex in our study community is a taboo subject, and gender and generational matters are major discussion issues. When discussion around sex happens, it is either amongst peers or elders advising the youth. Even so, the discussion is conducted in language coated with euphemisms, politeness, idioms, gestures, and symbols because explicit expression is considered disgraceful and disrespectful. Engaging in sex is referred to as “*hitting*”. Polite terms are used to refer to sex organs; female genitals are referred to as “*inkomo*” (the cow), or “*police*”. Terms used for the male genitals include “*the stick*”, “*the boy*”, “*the thing*”, “*the priest”*, and the *chimney*. The range of lexicon is used in a subversive way to camouflage sexual expression, a finding consistent with that of Buchanan-Aruwafu et al. [1] who detail the range of vocabulary that young people in Malaita in Solomon Islands use to disguise their expression of sex in a society where the subject is taboo and where one is subjected to severe punishment for breaking the social mores. Despite this apparent conservativeness around sexual behaviour, participants acknowledged the existence of causal as much as regular partners. 

The findings suggest that although sex is not discussed with partners, there are things that each gender does to enhance sexual pleasure. Notable is the fact that parents do not discuss sex with their children. This finding is consistent with traditional Zulu culture where parents did not discuss sex with their children because traditional institutions in the society ensured that sex education was provided by recognised women (*iqhikiza*) and men. The collapse of the traditional institutions that provided sex education to adolescents has left a vacuum that remains unfilled by the South African education system, yet parents still find it difficult to discuss sex with their children. The result is teenage pregnancy which is undesirable because of the disruption on the education of young women. It is notable that between 2001 and 2005 the rates of teenage pregnancy in this community declined to between 98 percent and 80 percent, respectively, [9] yet the high rates of teenage pregnancy point to gaps in sex education among adolescents. As elsewhere, the youth in South Africa receive their knowledge about sex from peers and the media (television, pornography, internet, etc.). Virginity in Zulu culture is valued and traditionally virginity testing is done to ensure that adolescent girls remain chaste. The reintroduction of this practice in KwaZulu-Natal province has been welcomed by some but has raised controversy amongst feminists because of its gender bias; it is conducted among girls only and not boys. The re-introduction of virginity testing is an attempt to deal with the contemporary challenges of the sexual behaviour of adolescent girls in a cultural way, yet this represents only a partial solution as the behaviour of the men remains unaddressed. 

In this study, the notion of casual partner was not verbalized but symbolized. The phenomenon of causal partners (*amakwapheni'*) arises out of the migrant labour system where male migrants to the cities only return after long intervals of up to a year in some instances [10] The phenomenon of causal partners is not only confined to this rural area but is common in South Africa. In a cross-sectional behavioural and HIV-1 seroprevalence survey among migrant couples in rural KwaZulu Natal, Lurie et al. [10] found evidence of casual partners for both the male and the female. The practice of casual partners in South Africa is highlighted in television shows such as *Isidingo* (award winning local soapie where the patriarch Zeb Matabane has a child with his casual partner Refiloe and his daughter, Lettie, dies in a traffic accident in disappointment after discovering that her husband, Vusi Moletsane, has a casual partner with Siyanda Mazibuko, who poses as a business associate). In *Soul city* (an educational programme on HIV/AIDS), a teenage orphan has her age mate as a regular partner and an older married man who provides her groceries as her casual partner. In *Emzini Wezinsinzwa* (programme about the life of male migrants living in Johannesburg hostels), Sophie is the casual partner of a Malawian migrant worker, Chirwane. 

Men who father children outside marriage are required to pay “damages” (*Inhlawulo*) to the family of the woman according to Zulu customary law. In the past, damages were paid in the form of cattle, but nowadays they are paid in terms of money, and the amount is set by the family of the girl. In addition to the cultural “damages”, the state requires fathers to provide maintenance for their children regardless of whether these are born within marriage or not. Where men fail to fulfil this obligation, they are sued for financial support for the child which then is deducted directly from their income. 

While sex is considered a taboo subject, the fact that society provides a way out for those involved in sex outside marriage and acknowledges the existence of casual partners presents a paradox. In a sense, it represents attempts by the society to deal with a contemporary phenomenon (extra-marital sex) in a cultural way, that is, through the payment of damages. The cultural and contemporary are intertwined in a complex way that may not be obvious to outsiders. Researchers would need to be open-minded not only to pick up these complexities but also to take them into account when conducting research on sexual behaviour in rural KwaZulu-Natal.

A range of traditional and modern methods are used by the men to enhance the sexual pleasure of their women. The traditional methods of enhancing sexual pleasure include doing enema, taking herbs and eating certain foods like eggs and cheese. The assertion that paying *Lobolo* helps enhance sexual pleasure should not be taken in the literal sense but should be understood in the sense that the man has peace of mind because he does not owe the family of his wife and he is assured that should his wife not comply, then customary law is on his side. In Zulu culture, a wife can be penalised for refusing to have sex with her husband [11]. In addition to the traditional methods of enhancing sexual pleasure, men also resort to modern methods such as buying a range of products from pharmacies which include *silver bullets*, *power rise*, *tiger*, and *tingdon*. The use of both traditional and modern methods of enhancing sexual pleasure suggests that rurality and modernity are interacting in a complex way in shaping the sexual behaviour of this rural community. Being rural does not exclude access to modern ways of enhancing sexual pleasure.

Most of the things that women do to enhance sexual pleasure are traditional, for example, using herbal medicine such as *dubulageqe* which grows in the area and can be readily harvested. However, concoctions such as *Bhekaminangedwa* have to be bought from *Muthi *traders who also dispense them together with a range of other herbs for various health problems. The *muthi* traders who dispense traditional medicine can be equated to the modern day pharmacists although some are also traditional doctors. Some of the practices both men and women use to enhance their sexual pleasure have implications for their reproductive health and HIV Prevention. Whereas using concoctions and herbs may only have an impact on the user, making incisions using unsterilized razors is a sure way of transmitting HIV. 

Consistent with existing literature [4, 6], people in this rural community use erotic movies and magazines as a means of enhancing sexual pleasure. What is done to enhance sexual pleasure in the community is strongly influenced by cultural practices, but urban or global forces through the availability of erotic movies and sex enhancing medicines are influencing sexual behaviour. 

The finding that oral sex is used to enhance sexual pleasure is consistent with findings on sexual behaviour in Laos [6]. There is however a difference. Whereas oral sex was cited as enhancing sexual pleasure by the men in rural KwaZulu-Natal, in Laos, oral sex was cited by the women and this can be attributed to the design of the Laos study that only interviewed young women, whereas our study focused on men and women from the general population. 

Whereas the Laos study noted that oral sex gave the men the feeling of dominance over the women, the same was not cited in our study on rural KwaZulu-Natal. However, the fact that oral sex was mentioned in the men's focus groups and not the women's may confirm the feeling of men's dominance as noted by the Laos women. Whereas vaginal sex “dog style” was mentioned as enhancing sexual pleasure among the men in rural KwaZulu-Natal, the literature is silent about it. 

Notable is the covert use of aids for enhancing sexual pleasure among both men and women. The covert use of traditional medicine is not unique to this community but a common phenomenon in African communities where traditional medicine is still practiced. It is common for people in this community as elsewhere in rural South Africa to visit traditional healers when they have problems with their spouses, children, or in-laws. Such visits are often covert, and the suggested solution may also be applied covertly. In this paper, *Bhekaminangedwa* is an example a concoction used by women on their wayward sexual partners. However, other herbs such as *dubulageqe* and *amahlanzolwandle* are known to be of general use in detoxifying the body even though in this study they were cited by as helping to enhance sexual pleasure. 

Reporting on sexual behaviour is gendered. Men dismissively suggested that questions about vaginal hygiene and what women do to enhance sexual pleasure should be referred to the women themselves. Women too suggested they know little about the things that men do to enhance sexual pleasure. Such attitudes may not necessarily reflect lack of knowledge but a preference not to speak to matters that can best be articulated by members of the opposite sex. Where the views of both the men and the women converged was in terms of what was done culturally to enhance sexual pleasure, for example, doing enema. Traditionally, young teenage girls were assigned to specific older women (*Iqhikiza*) for sex education. Similarly, young men got their knowledge about appropriate sexual behaviour from older men in the community. However, things have changed. Contemporary teaching of sex education does not necessarily impart culturally appropriate sexual mores and values.

## 6. Conclusion: Implications for Practice

This paper set out to discuss sexual behaviour in a rural community. The paper notes that in rural KwaZulu-Natal like elsewhere, sex is a taboo subject, and when it is discussed, a range of euphemisms metaphors and colloquialisms are employed. The paper argues that discussion about sexual behaviour elicits gendered responses. Further, what is done to enhance sexual pleasure is informed by cultural practice although the influence of urban or global trends is also reported. We posit that to understand sexual behaviour in this rural community is important, but to ignore the urban or global influences only provides a partial understanding of sexual behaviour here as elsewhere in rural South Africa. Sex education programmes need to build on what remains of the social institutions and cultural practices of the Zulu people such as the value placed on virginity and male circumcision and to integrate these in sex education.
